# Sustained Viral Response Hematological Markers During The Treatment of Chronic Hepatitis C Infection in Children

**DOI:** 10.5812/hepatmon.6256

**Published:** 2012-09-30

**Authors:** Oscar Garcia-Algar, Laura Garriga, Cristina Molera

**Affiliations:** 1 Paediatrics Unit, Hospital del Mar, Parc de Salut Mar, Barcelona, Spain

**Keywords:** Antibodies, Viral, Biomarkers, Pharmacological, Hepatitis C, Chronic, Child

Dear Editor,

We read with interest the paper by Pawlowska et al. (1) and we would like to add some comments. They conducted a retrospective cohort study of children with chronic hepatitis C (CHC) infection who completed treatment with ribavirin (RBV) plus pegylated IFN (peg-IFN) or recombinant IFN, and concluded that a mild decrease in hemoglobin levels , leukocyte and platelet counts during treatment may be the responsible factors for sustained viral response (SVR) induction. One of the interesting data in this paper is the non-discussed main result: SVR was achieved with a non-statistically significant different rate in the two groups of treated children (RBV plus peg-IFN or IFN). In a previous article by Pawlowska et al. (2), the authors concluded that treatment with peg-IFN and RBV was effective, showing the same SVR percentage that appears in the current article, but without comparing it to another group treated with recombinant IFN and RBV. Manns MP et al. (3) and other adult population studies showed that therapy with peg- IFN and RBV was better than monotherapy with IFN or therapy with IFN plus RBV. Current guidelines recommend children are treated for hepatitis C virus (HCV) using the same principles applied in adults. However, few published studies in the pediatric population for HCV treatment assessed the effectiveness of these two treatments. A systematic review of the literature by Hu J et al. (4) found only four randomized controlled trials (RCTs) and 31 non-randomized studies, all involve IFN, peg-IFN, or combinations of these drugs with RBV. The SVR rate could not be directly compared as the populations and interventions differed across studies. Nevertheless, the results were comparable to studies in adults. The therapy must be avoided in children younger than two years of age because of the risk of neurotoxicity. The decision of start therapy time must account for the estimated or known duration of infection, genotype, and degree of fibrosis. On the other hand, the main question discussed in the paper is if the decrease in hematological parameters could be a response marker or simply a side effect. Agreeing with some previous studies (4, 5), anemia during treatment with peg-IFN and RBV in patients with HCV genotype 1 is associated with superior virologic responses. The results show that hemoglobin value, leukocyte and platelet counts decrease in both groups, but hemoglobin level and leukocyte count decrease more in the peg-IFN one. The authors show that the reduction was lower in children who achieved SVR than in those who did not, and peg-IFN had a more marked influence on the decrease in hemoglobin value and leukocyte count. They also suggest that the amount of this decrease could be a marker of beneficial prognosis in children same as in adults and that it may not be indicator for withholding or reducing the dose of IFN. According to the results of this study, it seems reasonable to recommend a close monitoring of different hematological parameters during treatment of children with CHC including IFN plus RBV, as possible side effects and surrogate markers of treatment response. However, according to (4), we conclude that current guidelines for the treatment of HCV in children are based on limited data and further research is needed to define the optimal therapy for HCV in children.
